# Coil embolization of a thoracic aorta hematoma with branch artery pseudoaneurysm – case report

**DOI:** 10.1186/s42155-020-00128-z

**Published:** 2020-08-16

**Authors:** Lorant Szabo, Tomas Reyes del Castillo, David Benz, J. Roos, Robert Seelos, Ruben Lopez-Benitez

**Affiliations:** 1 Department of Radiology and Nuclear Medicine, Luzerner Kantonsspital, 6000 Luzern 16, Switzerland; 2 Department of Vascular Surgery, Luzerner Kantonsspital, 6000 Luzern 16, Switzerland

**Keywords:** Novel technique, Experimental treatment, Embolization, Aortic injury, Side branch pseudoaneurysm

## Abstract

**Background:**

A thoracic aorta hematoma with branch artery pseudonaneurysm is a very rare complication of thoraric blunt trauma. The standard treatment of this type of injury is aortic endograft placement.

**Case presentation:**

We present a case in which a thoracic aorta hematoma with branch artery pseudoaneurysm was treated with coil embolization instead of endografting.

**Conclusions:**

Coil embolization of aortic injuries may be a safe and definitive treatment alternative in selected cases. This technique has the potential to reduce the risk of procedure-related complications.

## Background

Thoracic aortic injuries are one of the most devastating consequences of thoracic trauma. Blunt thoracic trauma is the second most common cause of thoracic aortic injury, typically caused by high-speed motor vehicle accidents, sport injuries, or falls.1.5–2% of patients suffering blunt thoracic trauma develop an aortic injury and 70–80% of them die at the scene of the accident (Lundervall 1964). Aortic injuries associated with blunt trauma are classified according to the Society for Vascular Surgery (SVS) in 4 grades. Grade I injuries (intimal tear) are treated conservatively, while grade II to IV injuries (intramural haematoma, pseudoaneurysm and rupture) are typically treated with endograft placement or open surgery (Lee et al. 2011). However, aortic endograft placement may occasionally lead to severe complications, depending on the level of graft placement and the length of coverage. Paraplegia and paraparesis due to spinal cord ischaemia are the most severe complications of thoracic endovascular aortic repair (TEVAR) with an overall risk of 3.9% (1.1–13.3%) (Rizvi and Sullivan 2010; Bobadilla et al. 2013).

**Fig. 1:**
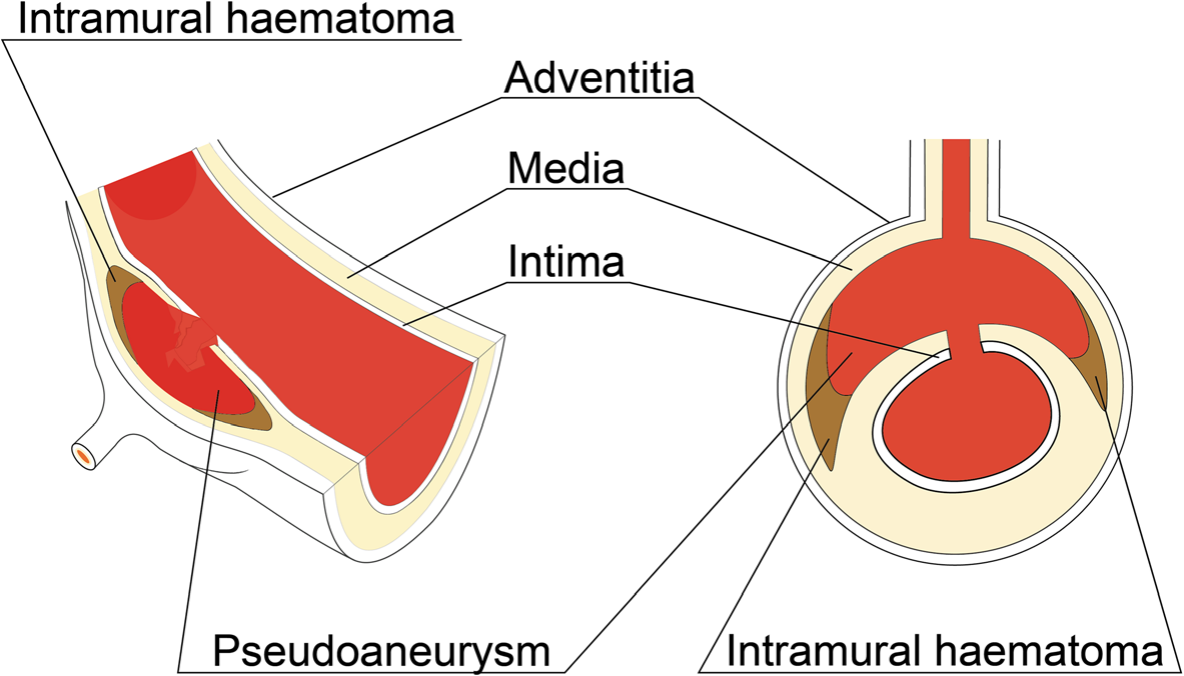
Diagram showing the mixed aortic injury (grade II and III) in two different perspectives. This lesion is characterized by a disruption of the intima and media layer with a contained pseudoaneurysm in the ostium of an intercostal artery

We present a patient in which a mixed aortic injury (grade II-III) (Fig. 1) was treated with a new approach using coil embolization instead of an aortic endograft.

## Case presentation

### Initial presentation

**Fig. 2:**
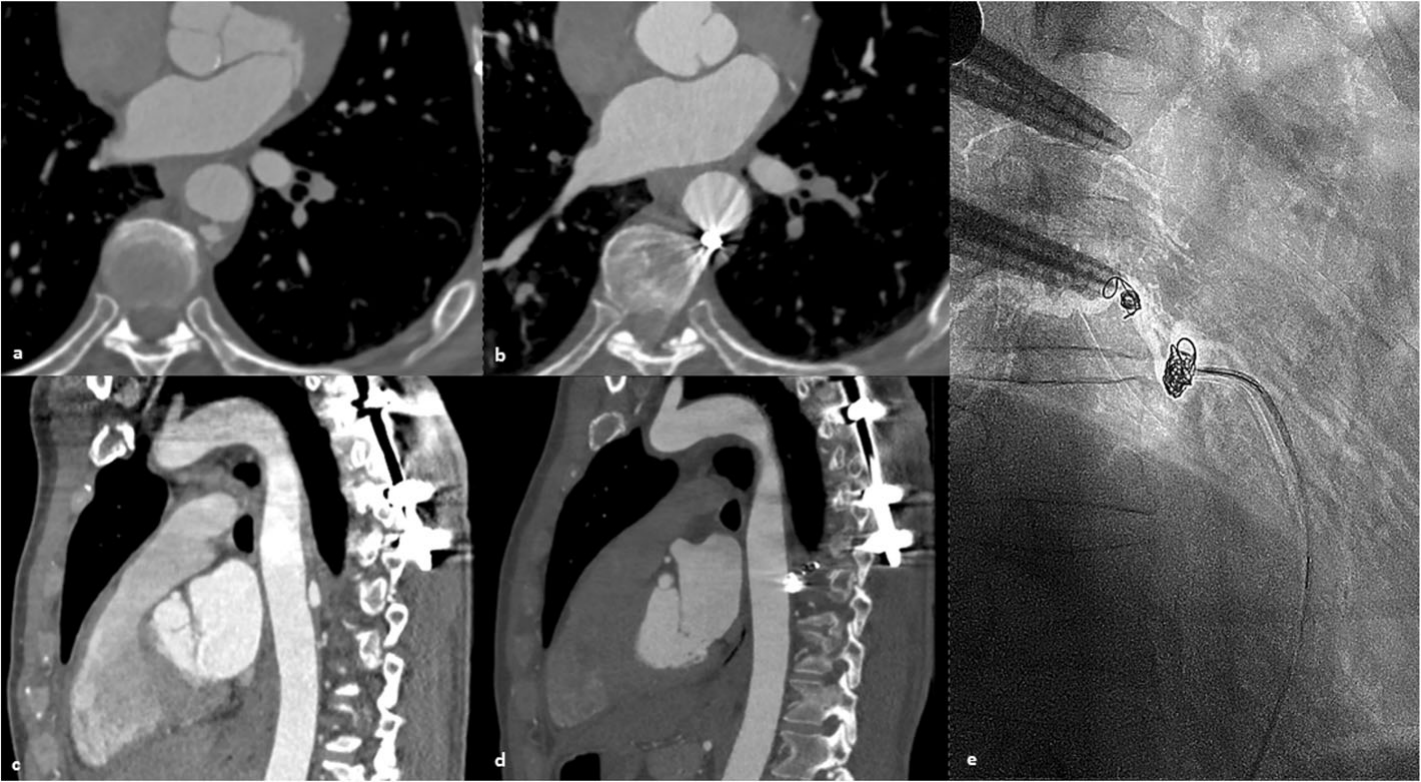
**a**, **b** Thoracic aortogram in oblique position shows pre and post treatment appearance of the aortic injury (grade II-III) treated with coil embolization

**Fig. 3:**
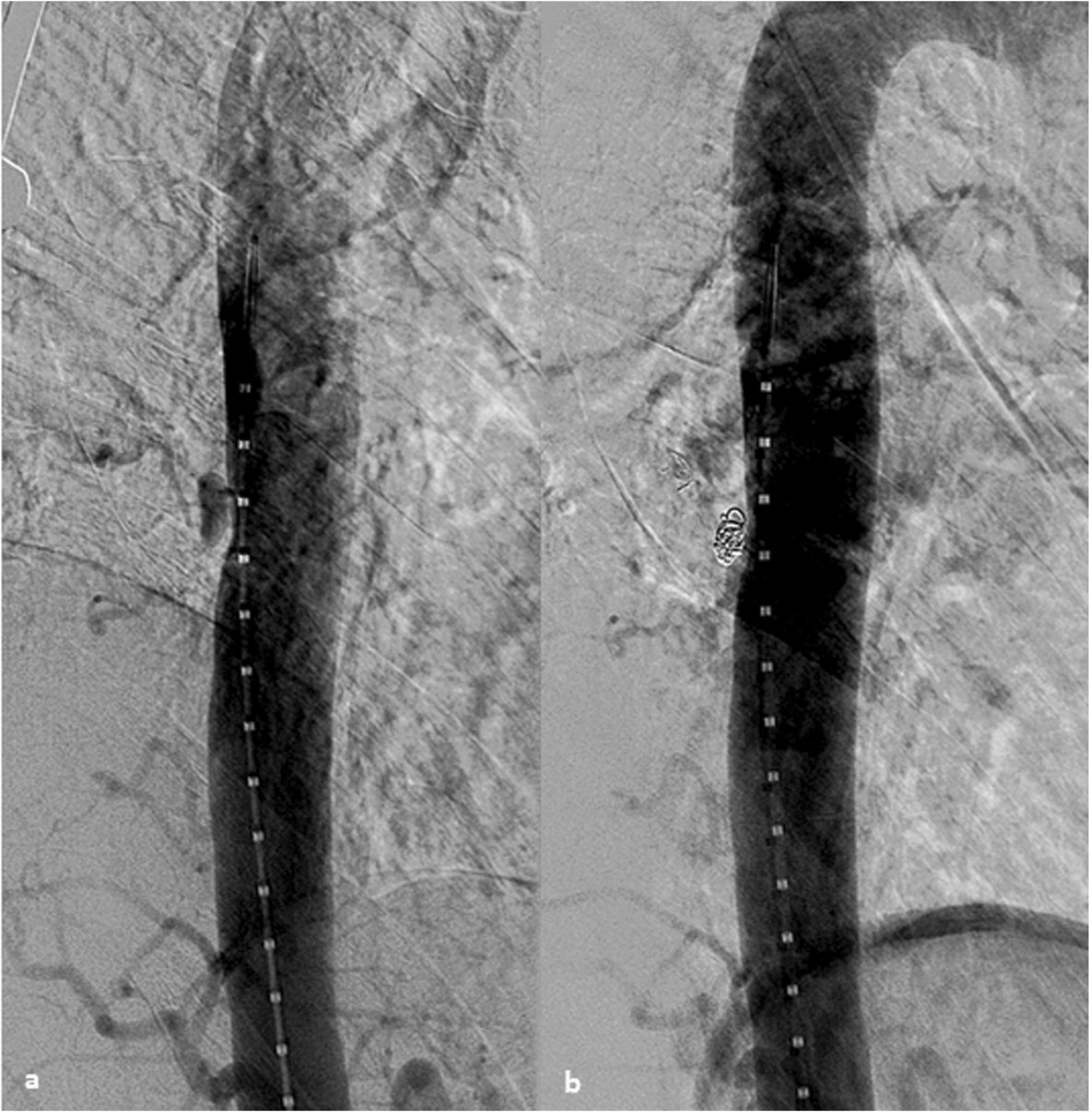
**a**, **c** Axial and sagittal images demonstrating intramural aortic haematoma with branch artery pseudoaneurysm at TH8 level. **b**, **d** Evidence of successful embolization after 21 months of follow, there are no signs of complication or re-filling of the pseudoaneurysm. **e** Flouroscopic image showing the coil-deployment inside the branch artery pseudoaneurysm

A 59-year old man with history of a sports-associated polytrauma was admitted to our hospital. The initial whole-body computer tomography (CT)-scan showed an aortic lesion at the level of TH8 in addition to multiple rib fractures and a TH6-vertebral fracture. The aortic injury presented with the typical features of intramural hematoma with contained blood collection between the sub-intimal layer and the muscular and adventitial layers. The aortic tear also caused a pseudoaneurysm in the ostium of the TH8-intercostal artery with the following characteristics: a size of 11 × 7 × 16 mm and a small intimal defect of 1.4 × 1.8 mm (Figs. 2 and 3).

Because we were concerned regarding a potential endoleak type 2 through the intercostal artery into the defect after endograft placement and the risk of medullary ischaemia, we decided to treat the lesion through coil embolization.

### Interventional procedure

After written informed consent by the patient, an elective intervention was performed under local anesthesia using a 4Fr right common femoral artery access.

A directed thoraco-lumbar (TH4-L2) aortogram was performed using a 4Fr straight graduated catheter (Angiodynamics Accu-Vu, Queensbury, NY, USA) (Fig. 3). Selective catheterization of the aortic tear at TH8 level was performed using a 4Fr SIM-1 catheter (Cordis, Miami Lakes, Florida, USA). Selective angiography confirmed communication of the cavity with the intercostal artery at the level of TH8. The spinal artery or anastomoses were not present between the TH8 intercostal artery and other vessels. We changed to a 4Fr C2 Cobra catheter (Cordis, Miami Lakes, Florida, USA) and using a 2,7Fr coaxial microcatheter system (Terumo Progreat, Tokyo, Japan) we selectively catheterized the corresponding intercostal artery TH8 via the aortic tear. First, a 10x320mm Hydrogel coated coil (Azur Microvention, Tustin, California, USA) was deployed in the intercostal artery to avoid future retrograde re-perfusion of the aortic lesion (back door embolization). Then, the pseudoaneurysm was filled with four 6x100mm IDC coils (Boston Scientific, Cork, Ireland). The coiling was challenging due to instability of the microcatheter, especially during the last phase of coil deployment, nevertheless, sufficient coil packing was achieved. The final angiogram showed complete occlusion of the pseudoaneurysm and no evidence of retrograde perfusion through the intercostal artery previously occluded (Fig. 2). No early complications were observed. The 4Fr sheath was removed, followed by manual compression. The total intervention time was 48 min from initial arterial access to last angiogram with a total radiation dose of 143,836 mGy/cm2.

### Follow-up

During and after the procedure, vital signs and neurological signs were clinically monitored (paresthesia, temperature, power and sensation in the lower limbs). There was no clinical evidence of acute spinal ischaemia. The patient was sent to an observation unit for 24 h and was discharged from the hospital afterwards. After 3, 6, 12 and 21 months, the patient was followed with CT-Angiograms that showed no extension of the aortic tear (Fig. 3). The implanted coils remained in place and the aortic wall was completely normal without signs of aortic lesion reperfusion or complications (i.e. aortitis, aneurysm, dissection). During all this time the patient remained asymptomatic.

## Discussion

Traditionally, aortic injuries have been treated with endovascular stent grafting or open surgical techniques (Pang et al. 2015). However, new minimally invasive techniques can be used to treat selected cases as in this case (Luebke and Brunkwall 2014). The endovascular approach makes the post-interventional recovery faster and potential complications unlikely (i.e. wound infections, blood loss). With modern fluoroscopy imaging systems, the radiation dose is low and localization of vascular complex lesions more feaseble to identify and treat (Daye and Walker 2018). The use of coils instead of an endograft reduced the risk of medullary ischemia and allowed us to perform the intervention in an outpatient setting under local anesthesia. Thus, other costs from a hospital stay, such as intensive care unit placement, general anesthesia and an expensive endograft are avoided (Azizzadeh et al. 2013).

From the technical point of view, the embolization, in this case, was possible because the aortic tear was contained and only communicated with the thoracic aorta through a small hole, measuring 1,3 mm. This situation makes a coil embolization safe because the risk of coil migration is smaller in comparison to bigger wall defects, where retaining the coils inside the lesion is often difficult. Another special technical aspect was the application of the front and back door embolization concept due to the communication with the intercostal artery. When pseudoaneurysms are embolized in the presence of inlet and outlet vessels, the embolization of the pseudoaneurysm alone is not recommended because of potential reperfusion through the feeder vessels (Madhusudhan et al. 2016; Salsamendi et al. 2016). In this particular case, the front door - meaning the branch artery ostium- was not embolized.

Branch artery pseudoaneurysms are usually self-limited with a benign clinical course, making conservative treatment a feasible approach. Nevertheless, in case of pseudoaneurysm growth or persisting back pain, endovascular embolization is a safe and effective alternative procedure (Ferro et al. 2013).

## Conclusions

In selected cases, mixed aortic injuries (grade II-III) may be eligible for coil embolization. This offers the possibility of a safe and less expensive treatment with reduced risk of procedure-related morbidity and complications.

## Data Availability

Not applicable.

## Abbreviations

SVS: Society of Vascular Surgery
TEVAR: Thoracic endovascular aortic repair
CT: Computer tomography
